# Shoulder contouring with botulinum toxin and fillers

**DOI:** 10.1016/j.jpra.2026.05.054

**Published:** 2026-06-03

**Authors:** Kyu-Ho Yi, Isabella Rosellini, Kang Hoon Choi, Han Earl Lee

**Affiliations:** a You and I Clinic, Seoul, Republic of Korea; b Avery Beauty Clinic And Avena Aesthetics, Indonesia; c Cheongdam Park Clinic, Seoul, Republic of Korea; d Opening Plastic Surgery Clinic, Republic of Korea

**Keywords:** Shoulder contouring, Botulinum toxin, Acromial filler, Clavicular filler, Aesthetic procedures

## Abstract

**Background:**

Interest in shoulder aesthetics has increased, with emphasis on clavicular definition and balanced shoulder contour. Minimally invasive approaches combining hyaluronic acid fillers and botulinum toxin type A (BoNT-A, Letibo, Hugel, Korea) have been proposed to address both structural and muscular components.

**Methods:**

This technical note describes three patients who underwent combined acromial and clavicular filler injections with BoNT-A administered to the upper trapezius and deltoid regions for shoulder contour refinement. Filler volume was individualized according to baseline shoulder slope, clavicular/acromial projection deficiency, soft-tissue thickness, and the degree of asymmetry, with total volumes of 10, 12, and 15 cc. BoNT-A was administered as 25 units distributed across five anatomically defined injection points. Outcomes were assessed by standardized clinical photography, clinical follow-up, patient-reported satisfaction, and adverse-event grading using the Common Terminology Criteria for Adverse Events framework.

**Results:**

All three patients demonstrated visible, qualitative improvement in shoulder contour, including enhanced clavicular definition and reduced trapezius prominence. Patient-reported satisfaction was favorable in all cases. No major complications were observed. Minor adverse events, including transient swelling and discomfort, were classified as mild CTCAE grade 1 events and resolved without intervention.

**Conclusions:**

This three-case technical note illustrates a feasible anatomy-guided multimodal approach for shoulder contour refinement. Because of the very small sample size, lack of a control group, and predominantly qualitative outcome assessment, these observations should not be interpreted as evidence of efficacy, safety, or generalizability. Larger controlled studies with standardized morphometric measurements, validated patient-reported outcome instruments, and longer follow-up are required.

## Introduction

Interest in shoulder aesthetics has increased as contemporary cosmetic trends emphasize upper-body definition, clavicular lines, and balanced shoulder proportions. Because outcomes depend on the relationship between bony landmarks, soft tissue, and muscle bulk, an anatomy-based approach is essential for safe filler placement and appropriate neuromodulator dosing.^1^

Hyaluronic acid (HA) fillers and calcium hydroxylapatite (CaHA) can enhance clavicular projection and acromial prominence, while botulinum toxin type A (BoNT-A) can reduce selected muscular bulk and refine the neck-shoulder line.^2^^,^^3^ Published evidence on combined shoulder-focused treatment remains limited. This technical note reports three illustrative cases treated with clavicular and acromial fillers combined with BoNT-A injections to the upper trapezius and deltoid regions, focusing on anatomy-guided planning, injection rationale, volume selection, short- to mid-term observations, and the limitations of small case-based evidence.

## Material and methods

**Fig. 1:**
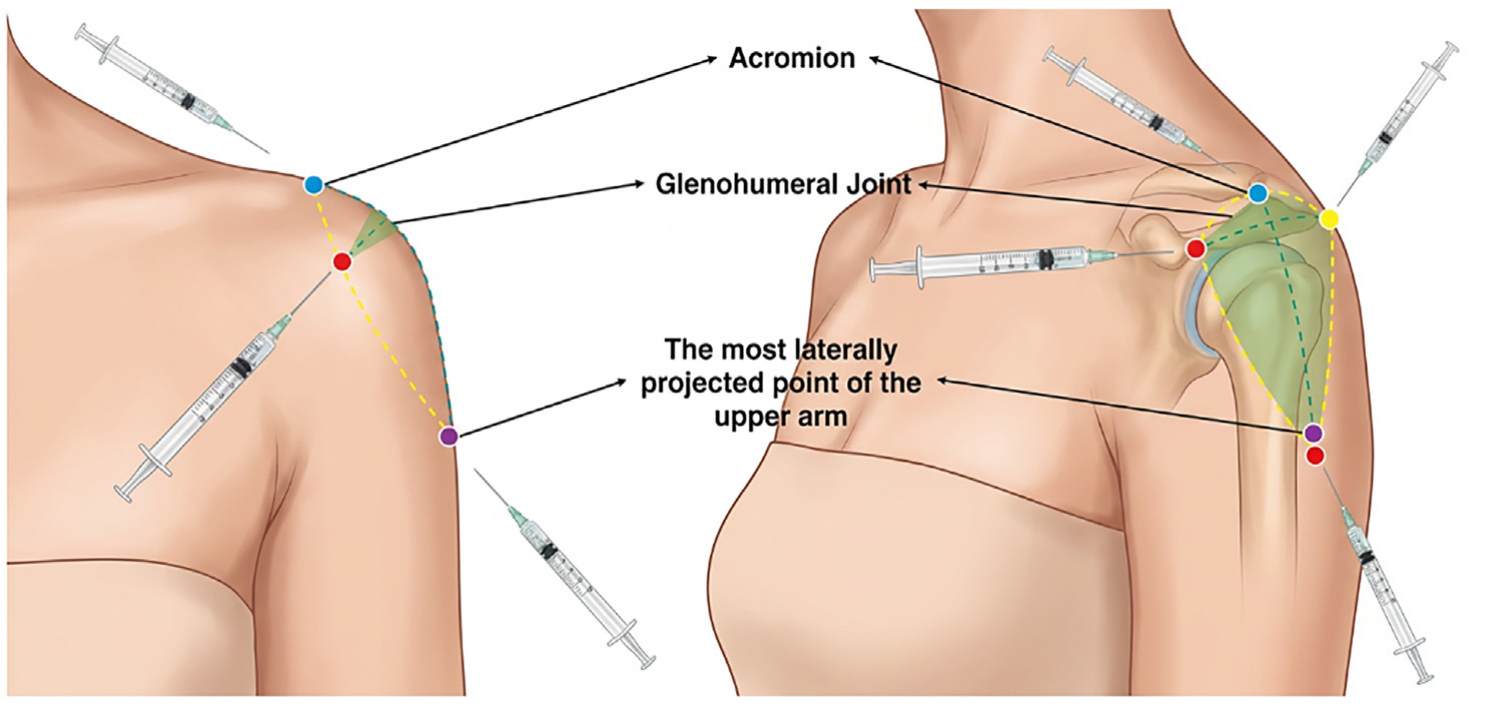
Anatomical landmarks and injection design for acromial and deltoid filler placement. The acromion, glenohumeral joint, and the most laterally projected point of the upper arm are identified to guide contour enhancement. Dashed lines illustrate the intended augmentation vectors and contour boundaries used for filler distribution.

This technical note included three patients seeking minimally invasive shoulder-contour improvement for trapezius prominence, reduced clavicular definition, mild acromial or lateral shoulder flattening, or imbalance of the neck-shoulder transition. Patients had realistic expectations and no contraindication to HA filler (Chaeum, Hugel) or BoNT-A (Letibo, Hugel) treatment, active local infection or inflammation, neuromuscular disorder, or major shoulder dysfunction. Filler injections were performed in the deep subcutaneous plane using a linear threading and fanning technique with the patient seated to preserve anatomical orientation and allow real-time assessment of the clavicular line, acromial projection, and shoulder slope (Fig. 1).

**Fig. 2:**
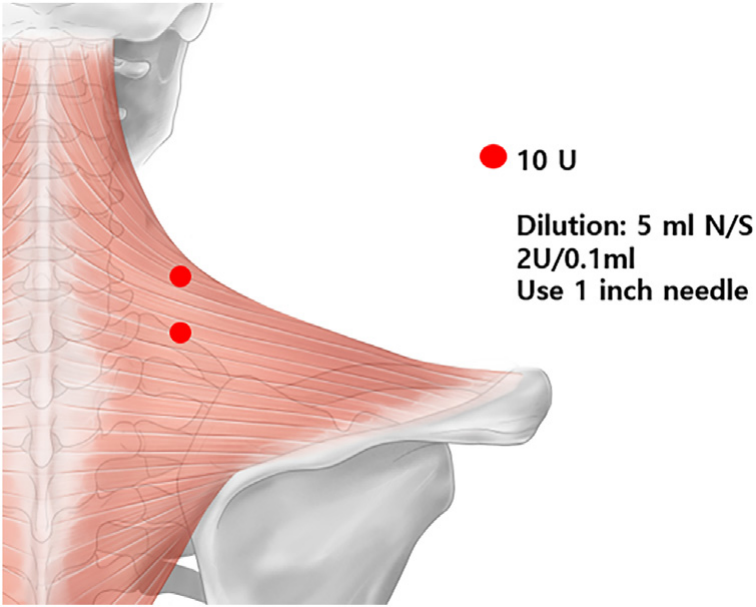
Injection mapping for botulinum toxin type A (BoNT-A) in the upper trapezius. The C7 spinous process and acromial landmarks are used to define the injection line. Five standardized injection points along the trapezius are shown, illustrating the distribution pattern used to achieve muscle modulation and contour refinement.

Local anesthesia was applied at entry points, with additional tumescent anesthesia used when needed for comfort. BoNT-A was administered as 25 units per patient, distributed as 5 units across five points. Injection mapping used reproducible surface landmarks, including the C7 spinous process, acromion, and visible upper trapezius contour, with points placed along the superior trapezius prominence to avoid overly inferior or deep placement. This approach was guided by prior neural arborization and accessory nerve distribution studies.^3^^,^^8^^,^^9^ Deltoid modulation was planned conservatively when lateral shoulder bulk contributed to contour Fig. 2.

Filler volumes were individualized at 10, 12, or 15 mL according to clavicular definition loss, acromial flattening, asymmetry, and shoulder-slope imbalance. Treatment was performed conservatively with repeated visual and palpation assessment, and injection was stopped when a smooth clavicular line, improved acromial transition, and acceptable symmetry were achieved. A 19G-21 G, 70 mm cannula was used, followed by gentle manual molding. Outcomes were assessed using standardized photographs, clinical follow-up, patient-reported satisfaction, and adverse-event review, including swelling, pain, bruising, nodularity, contour irregularity, infection, vascular compromise, weakness, and shoulder-motion limitation.

## Results

**Fig. 3:**
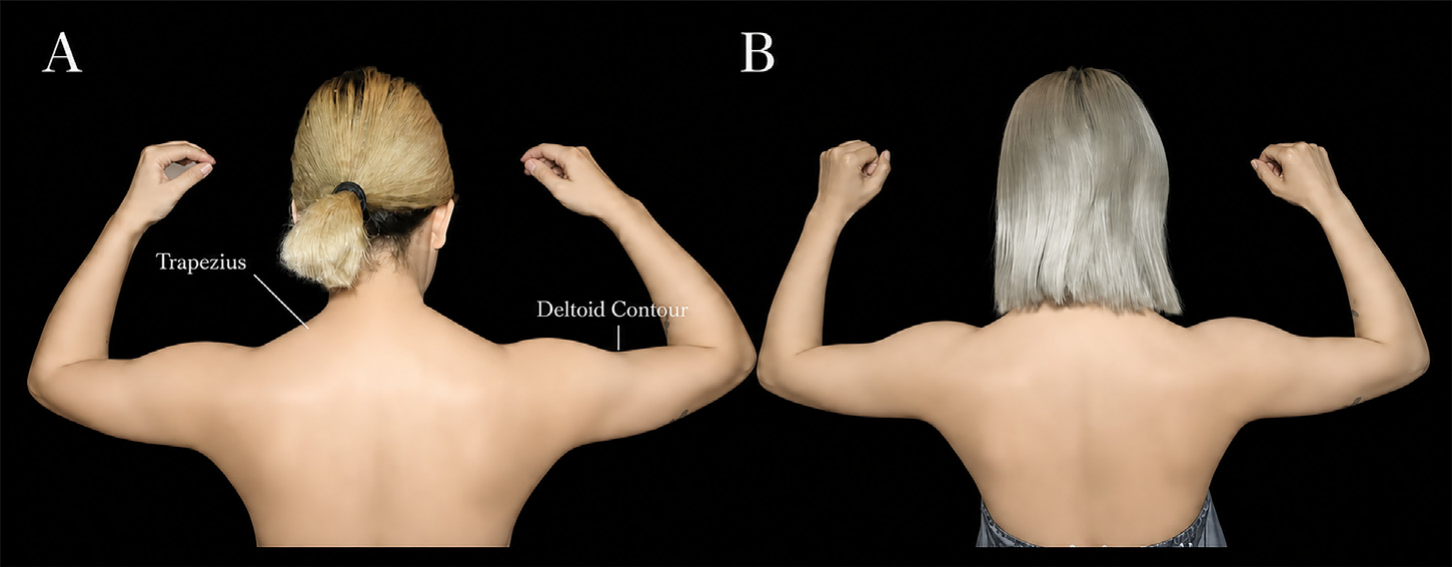
Clinical outcome in Case 1 following combined clavicular and acromial hyaluronic acid filler injection with BoNT-A treatment. Pre-treatment and post-treatment photographs demonstrate qualitative changes in shoulder contour, including reduced trapezius prominence and enhanced clavicular definition. The aesthetic outcome reflects subtle contour refinement rather than dramatic morphological change.

All three patients showed qualitative improvement in shoulder contour, including increased clavicular definition, smoother acromial transition, and reduced trapezius prominence (Fig. 3). All patients reported satisfaction with the post-treatment contour. Where follow-up photographs were available, improvement remained visible without clinically evident migration or delayed contour irregularity.

No standardized morphometric measurements or validated shoulder-specific aesthetic instruments were used. No major or long-term complications occurred. Minor swelling and discomfort at injection sites were mild, self-limited, and required no intervention. No infection, vascular compromise, nodularity requiring treatment, clinically significant shoulder weakness, or shoulder-motion limitation was observed.

## Discussion

Aesthetic refinement of the shoulder complex requires knowledge of clavicular, acromial, and upper-arm anatomy because these structures determine shoulder width, slope, and contour. The clavicle acts as the anterior strut of the shoulder girdle and provides key deltoid and trapezius attachments, particularly along its lateral third.1 Selective acromial and clavicular volumization may enhance the lateral shoulder apex and upper-body line, but filler placement should remain conservative and favor stable deep planes near relevant bony landmarks.

The present report describes a combined, anatomy-guided strategy using structural filler placement with BoNT-A neuromodulation. HA and CaHA fillers may improve clavicular projection, acromial transition, and deltoid-acromial smoothness, while BoNT-A may soften trapezius or selected deltoid contribution to contour.^2^, ^3^, ^4^ Previous reports have described clavicular filler contouring, deltoid and acromial augmentation, deltoid BoNT-A treatment, and trapezius BoNT-A contouring separately.^2^^,^^4^, ^5^, ^6^, ^7^, ^8^, ^9^ Our observations are consistent with these reports, including visible contour improvement and mainly transient local adverse events. However, the findings should be interpreted as preliminary clinical observations rather than evidence of treatment efficacy.

Safety depends on plane selection, landmark-based injection, and conservative dosing. Using the deep subcutaneous plane for acromial and deltoid filler may reduce vascular injury risk because major neurovascular structures, including the circumflex humeral arteries, are typically deep to the deltoid fascia. BoNT-A injection should avoid overly inferior or deep placement to reduce diffusion-related weakness or imbalance. The five-point upper trapezius design used here was selected to cover the visible neck-shoulder prominence while relying on reproducible C7 and acromial landmarks, consistent with anatomical studies of neural arborization and accessory nerve distribution.^3^^,^^8^^,^^9^ CaHA contouring may provide durable skeletal definition, but careful placement is needed to minimize migration, firmness, or nodularity.^10^

This technical note has important limitations. It included only three patients, had no control group, and used qualitative photographic assessment rather than standardized morphometric measurements or validated patient-reported outcome instruments. Filler volume also varied between patients, which limits procedural uniformity. Therefore, improvement cannot be causally attributed to the intervention alone, and the effects of positioning, lighting, placebo response, and observer bias cannot be excluded.

Overall, anatomy-guided integration of HA fillers, CaHA fillers, and targeted BoNT-A may offer a practical approach to individualized shoulder-contour refinement when performed conservatively. Larger studies with standardized protocols, objective measurements, longer follow-up, and formal safety assessment are required to clarify efficacy, durability, and reproducibility.This limitation is particularly relevant in aesthetic medicine, where patient perception and image standardization strongly influence outcome interpretation.

## Limitations

This technical note has several important limitations. First, the sample size was extremely small, consisting of only three patients, and the report therefore cannot establish efficacy, safety profiles, durability, or generalizability. Second, no control or comparison group was included; this is a fundamental methodological gap because causal attribution to the intervention cannot be confirmed. Third, outcomes were assessed mainly by qualitative clinical observation and photographic comparison, without standardized morphometric measurements such as shoulder angle, clavicular projection, acromial projection, or trapezius contour thickness. Fourth, validated patient-reported outcome instruments were not applied; patient satisfaction was recorded only as a simple categorical clinical assessment, and validated tools such as FACE-Q or a shoulder-specific aesthetic instrument should be incorporated in future studies. Fifth, filler volume varied between patients, although this reflected individualized anatomical planning rather than a standardized protocol. Finally, follow-up was limited, and longer-term evaluation is required to assess durability, delayed adverse events, migration, contour irregularity, and functional outcomes. Future studies should include larger cohorts, standardized injection algorithms, objective morphometric assessment, validated patient-reported outcome measures, systematic CTCAE-based adverse-event reporting, and appropriate control or comparison groups.

## Conclusion

This three-case technical note describes an anatomy-guided combined approach using acromial and clavicular fillers with BoNT-A injections for shoulder contour refinement. The observed qualitative improvements suggest that this multimodal strategy may be feasible in carefully selected patients. However, because of the very small sample size, lack of a control group, limited follow-up, absence of objective morphometric measurements, and limited patient-reported outcome assessment, these findings should be interpreted only as preliminary observations. Further controlled studies are required before efficacy, safety, durability, and reproducibility can be established.

## Declarations

### Funding

None.

### Ethical approval

Not required.

### Informed consent

Written informed consent was obtained from all participants for the procedure and for the use of de-identified clinical and imaging data.

### Financial disclosure

None of the authors has a financial interest in any of the products, devices, or drugs mentioned in this manuscript.

## Author contributions

All authors reviewed and approved the final manuscript. Conceptualization: Kyu-Ho Yi, Isabella Rosellini Writing—Original Draft: Kang Hoon Choi, Han Earl Lee

Writing—Review & Editing: Kyu-Ho Yi, Isabella Rosellini, Kang Hoon Choi, Han Earl Lee

## Declaration of competing interest

None declared.
